# Annotations

**Published:** 1901-11-16

**Authors:** 


### ANNOTATIONS.

### Electricity in Medicine.

Is it our innate conservatism, or our dread of quackery, or is it, perchance, the dampness of our climate that makes English practitioners so indifferent to electricity in the treatment of disease? It may be that each of these influences has something to do with it, but in any case the fact remains that in this country electricity is at a discount, while static electricity is practically unused. Our attention is drawn to the subject by the reports in the American medical journals of the annual meeting of the American Electro-Therapeutic Association which has just been held in Buffalo. Nothing particularly novel was brought forward at the meetings; it was a humdrum affair enough; but from the number of those who took part in the discussions, and the evident good faith and absolute confidence in their results displayed by the speakers, it is manifest that electricity occupies a position in the minds of practitioners in America which it does not occupy except in the minds of a small circle of specialists in England: and we may well ask whether this is entirely as it should be. No doubt electricity in every form and every degree can be had here. There are a few "institutions," there are perhaps three or four well-fitted electrical rooms at hospitals, and some of the specialists have installations; but we make bold to say that to the ordinary average medical mind in England, and to the ordinary average student as turned out from the schools, electricity means a trumpery little faradic machine, all beyond which is regarded as work for the specialist and as strongly tainted with quackery even at that. Wave currents, sinusoidal currents, cataphoric medication, *effluve*, and all the rest of the electrician's armamentorium, are mere jargon to the average practitioner. Yet these terms indicate means and methods which are in constant use both on the Continent and in America, and it seems high time that some pretty authoritative English pronouncement should be made regarding their utility. As it is it can hardly be denied that England is entirely out of the running in regard to the therapeutic uses of electricity. "And a good thing too!" we can imagine some people saying. But the position taken up by English medicine in regard to electricity ought to be the outcome of knowledge, not of ignorance, as we fear is now the case.

### The General Medical Council Election.

It is not easy to get up much interest in the coming election of direct representatives on the General Medical Council. Except in regard to the question of the midwives every reform is made to hinge on obtaining a preliminary reformation of the General Medical Council, and as it is agreed that this can only be done by aid of the leverage of a reconstituted British Medical Association, much of the discussion is outside the range of the practical. As to the midwives question it is not very likely that anything which the profession can do with this election will have any particular effect one way or the other. We do not say that if the medical profession, the thousands of practitioners scattered all over the country and in intimate touch with all sorts and conditions of men, could come to some decision in the matter, and could display some energy in enforcing its unanimous decision upon those by whom laws are made, the result would be without effect. On the contrary, we believe that if such an event could ever happen it would exercise a most momentous influence over the course of legislation. We see no signs, however, of any such unanimity or of any such enthusiasm. Indeed, the only signs of strong conviction come from the extremists, who, by advocating things that are quite impossible, neutralise even such influence as the more moderate members of the profession might be imagined to possess. So long as men maintain that midwifery belongs to medicine, so that those who practise it are intruding on the rights of medical practitioners, that the midwives are an "illegal class of persons practising illegally," and that the power of midwives to practise midwifery "was distinctly abolished by the Act of 1886," they are outside the whole discussion as it stands at the present time, and they might as well play skittles as talk about the question. It is by such extremists that the powers of the profession and the chance of its useful intervention in a question of serious moment are frittered away.

### Our Sanitary Dealings with Subject Races.

Reports from Havanna show with what characteristic thoroughness the Americans have tackled the sanitary problem with which they were confronted when they took possession of Cuba, and the great success which has attended their efforts points a moral which should be carefully considered by other countries having foreign possessions — especially perhaps by ourselves. Considering the vastness of our possessions, and the extremely varied habits, religion, and customs which obtain among our heterogeneous fellow subjects it is perhaps not to be wondered at that we have gone on the general principle of interfering with them as little as possible. But this principle has stood, and still stands as a serious bar to any improvement in the health condition of some of our greatest dependencies. The Americans, however, commenced their career as conquerors of foreign lands at a time when sanitary dogmas had already taken root. Hence they made no bones of imposing on Havanna a system of sanitation which somewhat astonished the inhabitants of that gay but deadly city. The results have been striking and show well what can be done, even when sewers are absent and paving is bad, by insisting on improvement in the interior hygiene of the houses. In his monthly report the chief sanitary officer for Havanna says "The constant house-to-house inspection has enabled this department to *force* the people to keep the interior of the houses in good condition." This is really the important point, and it is just in this that we fail in dealing with subject races owing to our adherence to the old principle of non-interference with castes, customs and religions. Are we right? More than 70 years ago we suppressed suttee, or at least we passed laws against it, and thought we had done a great and noble deed. But after all, what are a few widows—saved from the funeral pyre to lead a miserable outcast life—compared with the holocaust of victims sacrificed every year in India to the fetish of non-interference with native customs?

